# Devices with Tuneable Resistance Switching Characteristics Based on a Multilayer Structure of Graphene Oxide and Egg Albumen

**DOI:** 10.3390/nano10081491

**Published:** 2020-07-29

**Authors:** Lu Wang, Jinyi Wang, Dianzhong Wen

**Affiliations:** HLJ Province Key Laboratory of Senior-Education for Electronic Engineering, Heilongjiang University, Harbin 150080, China; 2171258@s.hlju.edu.cn (J.W.); wendianzhong@hlju.edu.cn (D.W.)

**Keywords:** nonvolatile memory, egg albumen, graphene oxide, conductive filament, memory device

## Abstract

We used graphene oxide (GO) and egg albumen (EA) to fabricate bipolar resistance switching devices with indium tin oxide (ITO)/GO/EA/GO/Aluminum (Al) and ITO/EA/Al structures. The experimental results show that these ITO/GO/EA/GO/Al and ITO/EA/Al bio-memristors exhibit rewritable flash memory characteristics. Comparisons of ITO/GO/EA/GO/Al devices with 0.05 ωt %, 0.5 ωt %, and 2 ωt % GO in the GO layers and the ITO/EA/Al device show that the ON/OFF current ratio of these devices increases as the GO concentration decreases. Among these devices, the highest switching current ratio is 1.87 × 10^3^. Moreover, the RESET voltage decreases as the GO concentration decreases, which indicates that GO layers with different GO concentrations can be adopted to adjust the ON/OFF current ratio and the RESET voltage. When the GO concentration is 0.5 ωt %, the device can be switched more than 200 times. The retention times of all the devices are longer than 10^4^ s.

## 1. Introduction

Natural biological materials have the advantages that they do not require artificial synthesis [1,2,3], they naturally degrade [4,5], and they show good compatibility with the body [6]. They have been applied in field-effect transistors [7], batteries [8], organic light-emitting diodes (OLEDs) [9], and resistive random-access memory (RRAM) [10,11,12]. The memory devices consisting of a nanocellulose-based resistive-switching layer and a nano-paper substrate exhibit non-volatile resistive switching with the capability of multilevel storage [13]. Sericin has been demonstrated to show resistance switching characteristics for non-volatile memory [14]. Silk fibroin can be used as a dielectric layer in resistive random access memory (RRAM), and traps near the conductive filaments have been confirmed by low frequency noise (LFN) measurements. These traps are involved in charge trapping and de-trapping [15]. For a device with an egg white dielectric layer, the current switching ratio can be increased by incorporating Au nanoparticles coated with SiO_2_ into the dielectric layer [16]. Memristors made of lignin on a flexible substrate can simulate synaptic behaviour. Similar properties have been observed using collagen extracted from fish skin [17,18]. Besides the devices based on cation migration, another major class of memristive devices is based on anion migration, especially oxygen anions [19,20]. 

Many organic polymers can be blended with carbon-based materials or combined into a multilayer structure to improve their performance. By adjusting the ratio of carbon nanotubes (CNTs) in composite polyvinyl alcohol (PVA)-CNT films, devices with bipolar switching and write-once-read-many (WORM) characteristics can be prepared [21]. The threshold voltage can be adjusted by varying the content of functionalized multi-walled carbon nanotubes (f-MWCNTs) embedded in poly(3,4-ethylenedioxythiphene) and sodium polystyrene sulfonate (PEDOT:PSS) [22]. By changing the RESET voltage in a polyimide (PI)/PI-graphene oxide (GO)/PI device, the device can be made to exhibit multilevel switching [23]. Regarding biological materials, semiconductor CdSe quantum dots can be mixed with silk protein to form the dielectric layer of a RRAM device with multilevel switching characteristics [24]. According to previous reports, GO has also been used as a material for RRAM. For composite GO-polymer materials, different GO mixing ratios will yield devices that exhibit adjustable resistance switching characteristics as a result of trapping and de-trapping of carriers in the GO [25,26,27,28,29,30]. Therefore, the electrical characteristics of devices can be modified by using various composite films or multi-layer structures. This can be explained by the mechanisms of carrier trapping and de-trapping [21,22,31], redox reactions induced by electric fields [23,32,33], and ion migration [24,34,35].

In the study reported in this paper, we fabricated indium tin oxide (ITO)/GO/egg albumen (EA)/GO/Al devices with GO concentrations of 0.05 ωt %, 0.5 ωt %, and 2 ωt % in the GO layers and an ITO/EA/Al device and analysed the effect of the GO concentration on these devices. The results show that the ON/OFF current ratio increases as the GO concentration in the GO layers decreases, while the RESET voltage decreases as the GO concentration decreases. In addition, when the GO concentration is 0.5 ωt %. The device can be continuously switched more than 200 times.

## 2. Materials and Methods

### 2.1. Materials and Device Fabrication

Eggs were purchased from a local supermarket and graphene oxide (GO) was purchased from Suzhou Hengqiu Graphene Technology Co., Ltd. (Suzhou, China). The purity of the GO was 96%, the thickness of the GO was approximately 1 nm, and the sheet diameter was 0.2–10 μm. GO was dissolved in deionized water at concentrations of 0.5 mg/mL, 5 mg/mL, and 20 mg/mL, which was followed by ultrasonication for 2 h to achieve uniform dispersions. Egg albumen (EA) and deionized water were mixed at a volume ratio of 1:8 (1 mL EA to 8 mL deionized water) and dispersed via ultrasonication for 25 min until being fully mixed. Indium tin oxide (ITO)-coated glass was sequentially washed with alcohol, acetone, and deionized water for 20 min each. A dielectric layer was formed via the spin coating method. A first GO layer was spin-coated at a spinning speed of 1000 rpm for 60 s and then placed in a drying box and heated at 100 °C for 30 min. An EA layer was spin coated on the GO layer at a low speed (500 rpm) for 5 s and then a high speed (4000 rpm) for 40 s and was then heated at 105 °C for 10 min. Lastly, a second GO layer was prepared under the same conditions as the first layer. We also used the same preparation conditions for the EA layer when making the ITO/EA/Al device. Lastly, a shadow mask (2 cm × 2 cm) was used to deposit Al on the dielectric layer via thermal evaporation to obtain a top electrode (consisting of many circular pads with a diameter of 1.0 mm) with a thickness of approximately 200 nm.

### 2.2. Characterization

The GO microstructure was observed with a transmission electron microscope (TEM, JEM-2100) (JOEL, Tokyo, Japan). A scanning electron microscope (SEM, Hitachi S3400) (Hitachi, Tokyo, Japan) was used to observe the cross section of the ITO/glass substrate coated with EA fluid. The ultraviolet-visible (UV-Vis) spectra of the EA film and the GO film were also measured by using a ultraviolet-visible (UV/VIS) spectrophotometer (UV/VIS, TU-1901) (Puxi, Beijing, China). Raman spectroscopy of GO and EA was performed by using a Thermo Fisher Scientific DXR system (Raman, DXR2xi) (Thermo Fisher Scientific Inc., Waltham, MA, USA) under excitation light of 532 nm. The changes in the functional groups in GO of different concentrations were measured by X-ray photoelectron spectroscopy (XPS, ESCALAB 250 Xi) (Thermo Fisher Scientific, Waltham, MA, USA). The zeta potentials of the different concentration GO solutions were measured by a nanoparticle size potential analyser (Zetasizer Nano, ZS90) (Malvern, Worcestershire, UK). The thickness and roughness of the GO layers were observed by atomic force microscopy. (AFM, Innova) (Bruker Corporation, MA, USA). The electrical properties of the ITO/GO/EA/GO/Al and ITO/EA/Al memristors were tested using a semiconductor parametric tester (Keithley 4200) (Keithley, Solon, OH, USA).

## 3. Results

**Figure 1:**
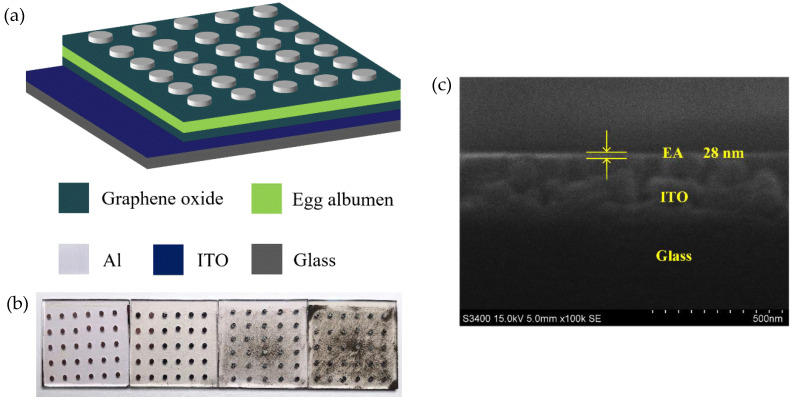
(**a**) ITO/GO/EA/GO/Al device structure. (**b**) Picture of ITO/EA/Al and ITO/GO/EA/GO/Al devices (from left to right are the ITO/EA/Al device and the ITO/GO/EA/GO/Al devices with GO concentrations of 0.05 ωt %, 0.5 ωt %, and 2 ωt % in the GO layers). (**c**) SEM cross-sectional image of an EA film.

**Figure 2:**
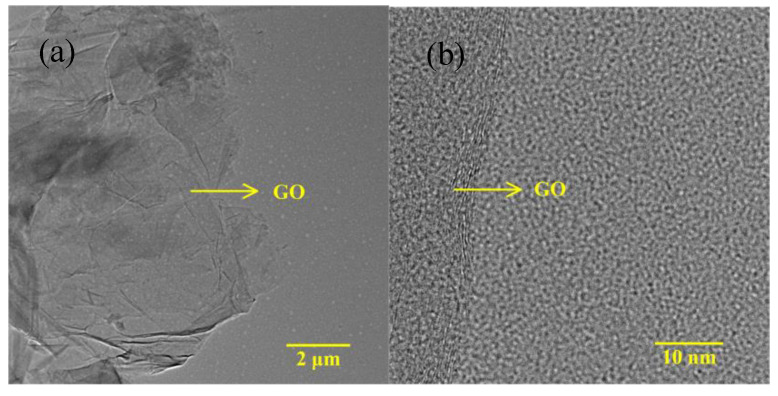
TEM images of GO: (**a**) low resolution and (**b**) high resolution.

**Figure 3:**
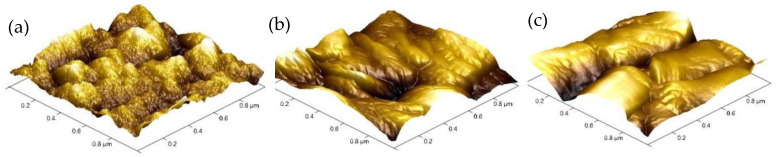
AFM images of GO films covering ITO glass with GO concentrations of (**a**) 0.05 ωt %, (**b**) 0.5 ωt %, and (**c**) 2 ωt % in the GO layers.

Figure 1a shows the Al/GO/EA/GO/ITO device structure. Figure 1b shows a picture of the ITO/EA/Al and ITO/GO/EA/GO/Al devices. The four devices in the picture from left to right are the ITO/EA/Al device and the ITO/GO/EA/GO/Al devices with GO concentrations of 0.05 ωt %, 0.5 ωt %, and 2 ωt % in the GO layers. An SEM cross-sectional image of an EA film is shown in Figure 1c. From bottom to top, the materials are glass, ITO, and the EA film, and the thickness of the EA film is approximately 28 nm. The GO microstructure was also observed at various resolutions via TEM, as shown in Figure 2. AFM images of GO films covering ITO glass with GO concentrations of 0.05 ωt %, 0.5 ωt %, and 2 ωt % in the GO layers are shown in Figure 3. The roughness of the GO film covering ITO glass with a GO concentration of 0.05 ωt % is 1.89 nm, which indicates that the interface is smooth and that the GO layer has a good interface contact with the ITO electrode. The smooth surface is beneficial to reduce the barrier between the active layer and the electrode. The roughness of the GO film covering ITO glass with a GO concentration of 0.5 ωt % and 2 ωt % is 18.8 nm and 9.73 nm, respectively, which indicates that, with increasing GO concentration, some GO agglomerates or folds appeared on the surface, but the roughness was not significant and had little impact on the electrical properties of the devices. The corresponding average heights of the GO films covering ITO glass with GO concentrations of 0.05 ωt %, 0.5 ωt %, and 2 ωt % in the GO layers are 31.22 nm, 124.24 nm, and 370.59 nm, respectively. With increasing GO concentration, the thickness of the GO layer increases. It has been reported that the thickness of the GO layer has little effect on the high-resistance state (HRS) and low-resistance state (LRS) [36].

**Figure 4:**
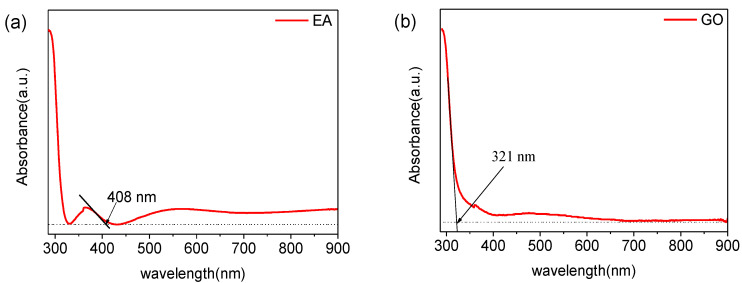
UV–Vis spectra of (**a**) the EA film and (**b**) the GO film.

We performed UV-Vis absorption spectroscopy on the EA film and the GO film, as shown in Figure 4. An ITO-coated glass sample was used for baseline correction. The edge of the absorption peak of the EA film is located at a wavelength of 408 nm, and the edge of the absorption peak of the GO film lies at 321 nm. According to the formula Eg = hc/λ, the band gap width of EA is 3.06 eV, and the band gap width of GO is 3.86 eV.

**Figure 5:**
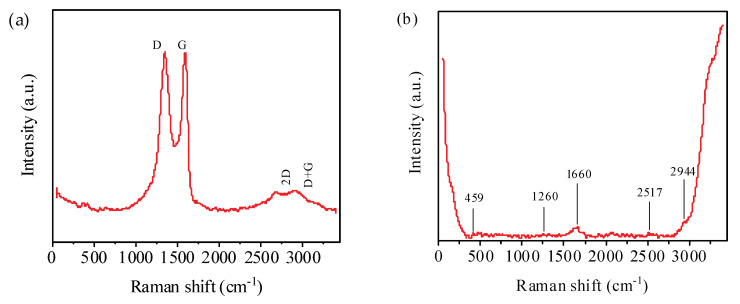
Raman spectra of (**a**) GO and (**b**) EA.

Figure 5 shows the Raman spectra of GO and EA measured with a 532-nm laser. In Figure 5a, the Raman spectrum of GO shows two well-defined bands at 1589.86 cm^−1^ (G band) corresponding to the stretching vibrations of sp^2^ carbons and at approximately 1349.55 cm^−1^ (D band), which indicates the disorder degree of the graphene structure [37]. The D band in the figure is slightly stronger than the G band and I_D_/I_G_ is approximately 1.003, which indicates that the defect density of GO is relatively high. A 2D peak was observed at 2684 cm^−1^, which indicates the presence of multilayers, and a D + G peak was observed at 2908 cm^−1^. This indicates a large number of defects in the GO sample [38,39,40,41]. Figure 5b shows the measured Raman spectrum of EA. The in-plane stretching vibration of the carboxyl group appears at 459.53 cm^−1^. The characteristic peaks at 500~1750 cm^−1^ represent proteins. The N-H bending vibration peak of the amide III band appear at 1260.33 cm^−1^. The absorption peak corresponding to 1660.30 cm^−1^ is attributed to the C=O stretching vibration of amide I. The absorption peak corresponding to 2517.20 cm^−1^ is attributed to S–H contraction. The stretching vibration peak of hypomethyl appears at 2944.69 cm^−1^.

**Figure 6:**
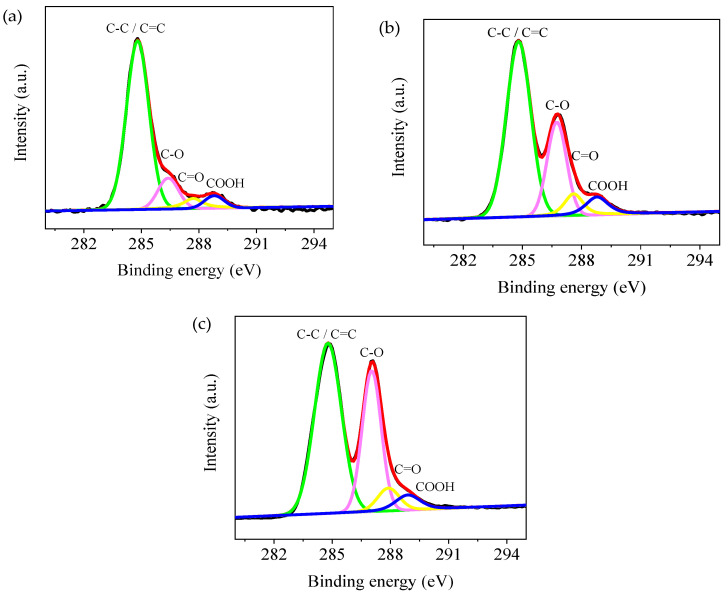
XPS spectra of C 1 s of the GO layers with (**a**) 0.05 ωt %, (**b**) 0.5 ωt %, and (**c**) 2 ωt % GO.

To study the chemical composition variation of the different concentrations of GO layers, XPS spectra were acquired. Figure 6 depicts the XPS spectra of C 1 s of the GO layers with 0.05 ωt %, 0.5 ωt %, and 2 ωt % GO. Four peaks are observed at 284.8, 286.4, 287.7, and 288.8 eV, which corresponds to C=C/C–C in aromatic rings, C–O (epoxy and alkoxy), C=O (carbonyl), and COOH (carboxylic) groups, respectively. With increasing GO concentration, the intensities of the C 1 s XPS peaks of epoxy and alkoxy gradually increase. This indicates that the concentration of oxygen-containing functional groups in the GO layer increases with a growing GO concentration. 

**Figure 7:**
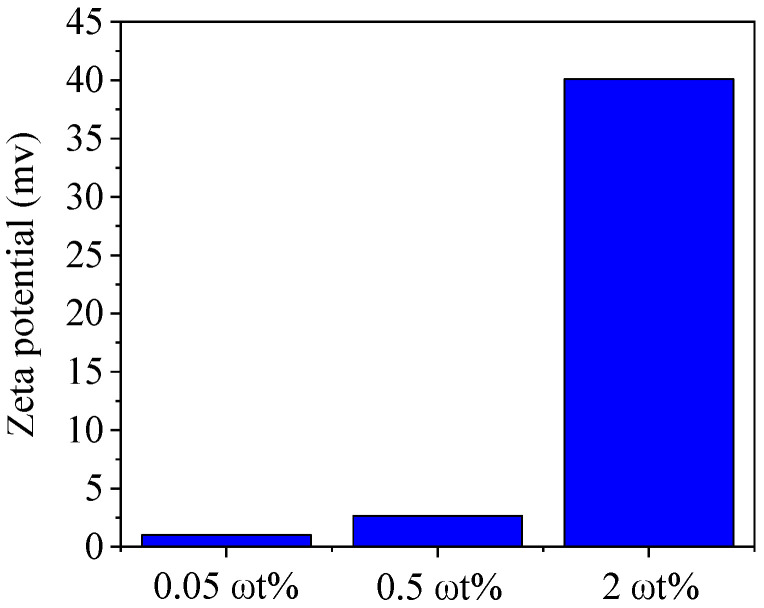
Zeta potential of GO suspensions of different concentrations.

To study the stability of GO solutions of different concentrations, the zeta potential was measured and analysed. Figure 7 shows the results of zeta potential detection for GO solutions with concentrations of 0.05 ωt %, 0.5 ωt %, and 2 ωt %. The figure shows that the stability of the solution increases with a growing GO concentration. The GO solution with a concentration of 2 ωt % is highly stable, up to approximately −40 mV.

**Figure 8:**
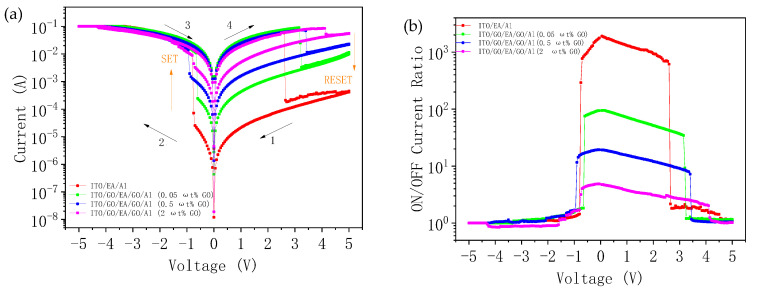
(**a**) I-V characteristics of ITO/EA/Al and ITO/GO/EA/GO/Al devices. (**b**) ON/OFF current ratios of ITO/EA/Al and ITO/GO/EA/GO/Al devices.

The typical I-V characteristics of the ITO/EA/Al device and the ITO/GO/EA/GO/Al devices with GO concentrations of 0.05 ωt %, 0.5 ωt %, and 2 ωt % in the GO layers are shown in Figure 8a. All the devices exhibit bi-stable switching characteristics. During the test, we set the sweep voltage range to be from −5 V to 5 V, and the bottom electrode (ITO) was grounded. The initial state of the ITO/EA/Al device was an HRS. When voltages of 5 V to 0 V were applied to the top electrode (Al), the device remained in the HRS. Then, we applied voltages of 0 V to −5 V to the device. When the voltage reached Vset = −0.75 V, the device transitioned from the HRS to an LRS, and when the voltage was then swept from −5 V to 0 V, the device remained in the LRS. Lastly, a voltage sweep of 0 V to 5 V was applied to the device, and the device switched from the LRS to the HRS when the voltage reached Vreset = 2.60 V. For the ITO/GO/EA/GO/Al devices with different GO concentrations, the same voltage sweep method as for the ITO/EA/Al device was used. Note that the introduction of GO with different concentrations did not change the polarity of the devices. The Vset voltages of the three ITO/GO/EA/GO/Al devices with GO concentrations of 0.05 ωt %, 0.5 ωt %, and 2 ωt % were found to be −0.60 V, −0.90 V, and −0.75 V, respectively, and the Vreset voltages were 3.20 V, 3.40 V, and 4.10 V, respectively, from which it can be concluded that Vreset decreases with a declining GO concentration. The ON/OFF current ratios of the ITO/EA/Al and ITO/GO/EA/GO/Al devices are shown in Figure 8b. The ON/OFF current ratio of the ITO/EA/Al device at 0.1 V was 1.87 × 10^3^. For the ITO/GO/EA/GO/Al devices with GO concentrations of 0.05 ωt %, 0.5 ωt %, and 2 ωt % in the GO layers, the ON/OFF current ratios at 0.1 V were 95, 19, and 5, respectively. We can conclude that, as the GO concentration decreases, the ON/OFF current ratio of the device gradually increases.

**Figure 9:**
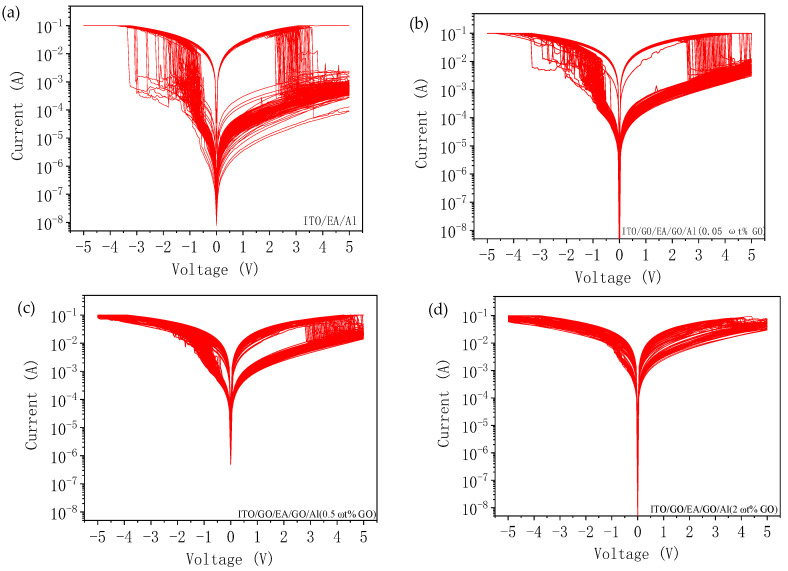
I-V characteristics under continuous cyclic scanning: (**a**) ITO/EA/Al device. (**b**–**d**) ITO/GO/EA/GO/Al devices with GO concentrations of (**b**) 0.05 ωt %, (**c**) 0.5 ωt %, and (**d**) 2 ωt %.

To study the continuous switching characteristics of the devices, we performed continuous cyclic scanning of the I-V characteristics of the ITO/EA/Al device and the ITO/GO/EA/GO/Al devices, as shown in Figure 9. The maximum number of times that the ITO/EA/Al device could be continuously switched was 110, and the ITO/GO/EA/GO/Al device with a GO concentration of 0.05 ωt % in the GO layers could be continuously switched a maximum of 100 times. The ITO/GO/EA/GO/Al device with a GO concentration of 0.5 ωt % could be continuously switched more than 200 times, whereas the maximum number of times that the device with a GO concentration of 2 ωt % could be continuously switched was 53. Based on a comparison of the above four devices, only the ITO/GO/EA/GO/Al device with a GO concentration of 0.5 ωt % in the GO layers showed an increase in the maximum number of switching cycles when compared with the ITO/EA/Al device without GO layers.

**Figure 10:**
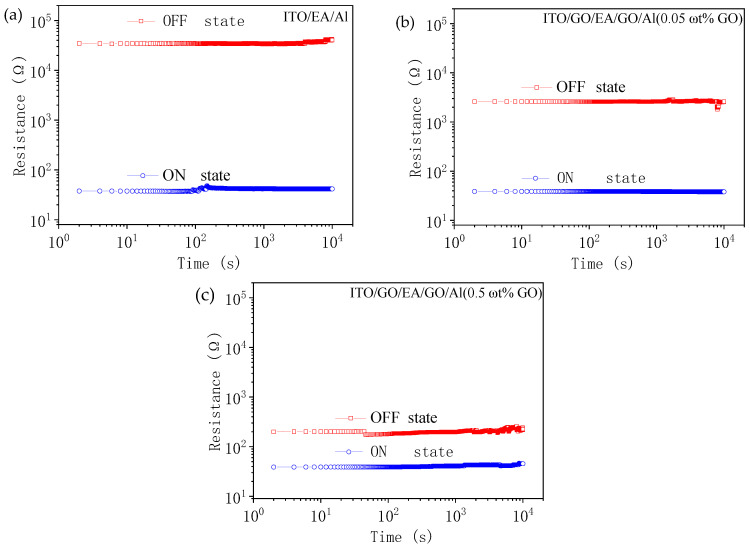
Retention times of (**a**) the ITO/EA/Al device and (**b**,**c**) the ITO/GO/EA/GO/Al devices with GO concentrations of (**b**) 0.05 ωt % and (**c**) 0.5 ωt %.

**Figure 11:**
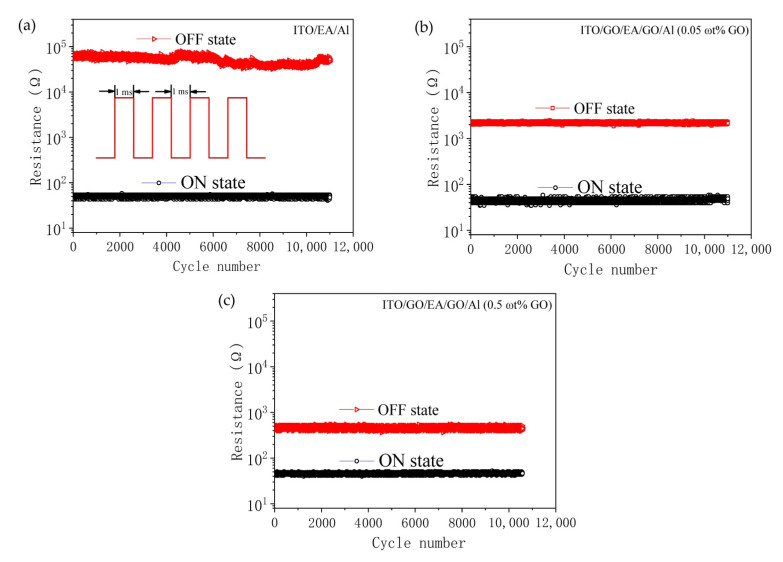
Endurance cycles of (**a**) the ITO/EA/Al device and (**b**,**c**) the ITO/GO/EA/GO/Al devices with GO concentrations of (**b**) 0.05 ωt % and (**c**) 0.5 ωt %.

We investigated the retention time and endurance of the devices by applying a constant voltage of 1 V and reading out data at this voltage to test the HRS and LRS retention characteristics. As shown in Figure 10, the retention times of all the devices were longer than 10^4^ s at this constant voltage, and the data did not degrade, which indicates that the devices have the ability to store data for a long time. The endurance characteristics of the devices were measured by applying a pulse with a period of 2 ms, a width of 1 ms, and an amplitude of 0.5 V. As shown in Figure 11, all the devices still had a clear storage window after 10^4^ continuous pulses, which indicates that the devices show reliable endurance.

**Figure 12:**
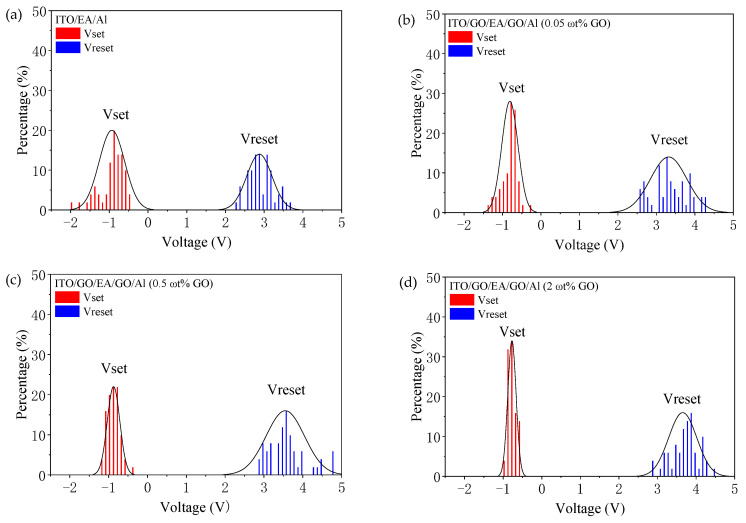
Vset and Vreset distributions of the devices: (**a**) ITO/EA/Al device. (**b**–**d**) ITO/GO/EA/GO/Al devices with GO concentrations of (**b**) 0.05 ωt %, (**c**) 0.5 ωt %, and (**d**) 2 ωt %.

To analyse the reliability of the devices, we summarized the I-V characteristics for the first 50 cycles of the ITO/EA/Al device and the ITO/GO/EA/GO/Al devices with GO concentrations of 0.05 ωt %, 0.5 ωt %, and 2 ωt % in the GO layers. The distributions of the SET and RESET voltages are plotted as histograms in Figure 12. The median SET voltage of the ITO/EA/Al device is −0.85 V, and those of the ITO/GO/EA/GO/Al devices with 0.05 ωt %, 0.5 ωt %, and 2 ωt % GO are −0.75 V, −0.85 V, and −0.80 V, respectively. The uniformity of the Vset distributions was then evaluated by calculating the standard deviations. The standard deviation of the SET voltage distribution of the ITO/EA/Al device is 0.327 V, and those of the ITO/GO/EA/GO/Al devices with 0.05 ωt %, 0.5 ωt %, and 2 ωt % GO in the GO layers are 0.210 V, 0.161 V, and 0.107 V, respectively. This result indicates that the higher the GO concentration is, the smaller the standard deviation of Vset is, which indicates that the uniformity of the Vset distribution increases with growing GO concentration. Meanwhile, the median RESET voltages of these devices are 2.80 V, 3.25 V, 3.50 V, and 3.70 V, which indicates that the RESET voltage increases as the GO concentration increases.

**Figure 13:**
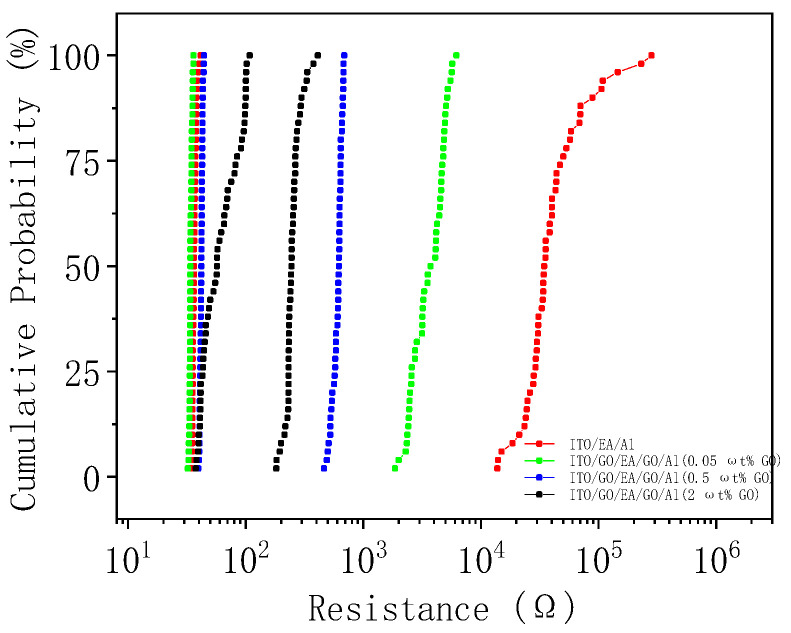
Cumulative probability of resistance for the ITO/EA/Al and ITO/GO/EA/GO/Al devices.

In Figure 13, the resistances in the LRS and HRS at 1 V are plotted as cumulative probability graphs. The median R_LRS_ of the ITO/EA/Al device is 36.12 Ω, and those of the ITO/GO/EA/GO/Al devices with GO concentrations of 0.05 ωt %, 0.5 ωt %, and 2 ωt % in the GO layers are 42.14 Ω, 42.25 Ω, and 57.01 Ω, respectively. The corresponding median R_HRS_ values are 2.02 × 10^4^ Ω, 4.59 × 10^3^ Ω, 6.21 × 10^2^ Ω, and 2.45 × 10^2^ Ω. Moreover, the coefficients of variation of R_LRS_ are 0.038, 0.025, 0.024, and 0.360, respectively. It can be concluded that the R_LRS_ values of the ITO/GO/EA/GO/Al device containing 2 ωt % GO are not as uniform as those of the other three devices. Lastly, the coefficients of variation of R_HRS_ are 0.973, 0.395, 0.091, and 0.168, respectively. By comparing the R_HRS_ and R_LRS_ results, we can see that the coefficients of variation of R_HRS_ are larger than those of R_LRS_, which indicates that the R_LRS_ distributions are more uniform and that the resistance distributions of the ITO/GO/EA/GO/Al device with a GO concentration of 0.5 ωt % in the GO layers are more uniform than those of the other devices.

**Figure 14:**
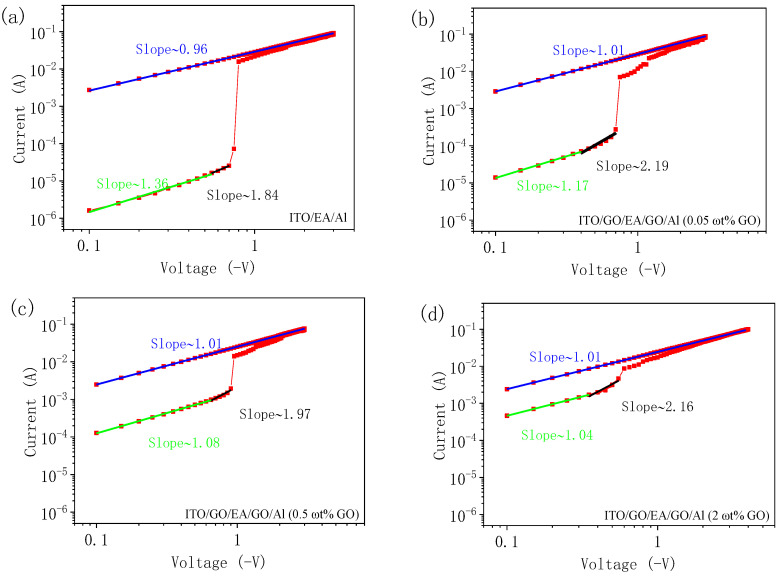
Log-log fits of the I-V curves at a negative bias: (**a**) ITO/EA/Al device, (**b**–**d**) ITO/GO/EA/GO/Al devices with GO concentrations of (**b**) 0.05 ωt %, (**c**) 0.5 ωt %, and (**d**) 2 ωt %.

The typical I-V characteristic curves in the negative bias region for all the devices were redrawn in log-log coordinates to analyse the carrier transport mechanism. A fitting analysis of the negative bias region for all the devices is shown in Figure 14. For the ITO/EA/Al device and the ITO/GO/EA/GO/Al devices with GO concentrations of 0.05 ωt %, 0.5 ωt %, and 2 ωt % in the GO layers, the fitted slopes of the I-V characteristic curves in the LRS are 0.96, 1.01, 1.01, and 1.01, which means that these devices exhibit ohmic behaviour in the conduction region. For the fitted I-V characteristic curves in the HRS when these devices are turned off, the slopes in the low voltage range are 1.36, 1.17, 1.08, and 1.04. The carrier conduction behaviour of the devices in this region still conforms to Ohm’s law. As the voltage increases, the fitted slopes gradually grow to 1.84, 2.19, 1.97, and 2.16. This process is consistent with the theory of the trap-controlled space charge current limiting mechanism [5].

**Figure 15:**
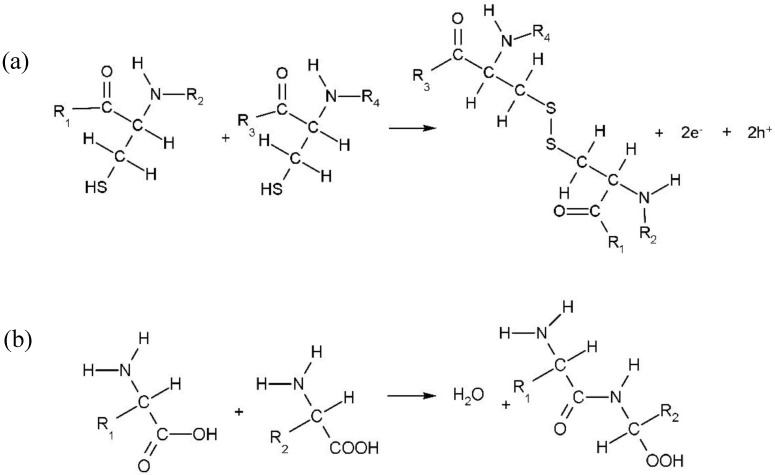
(**a**) Peptide bond and (**b**) disulphide bond generation process.

**Figure 16:**
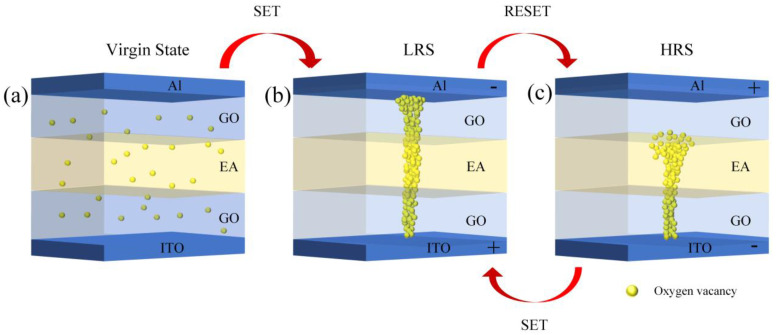
Model of the resistance switching mechanism.

Water is the main component of EA. Excluding water, nearly 90% of EA is composed of a series of proteins, such as ovotransferrin, ovalbumin, and ovomucoid [42]. EA becomes denatured when it is heated and peptide bonds and disulfide bonds are formed during the denaturation process, which causes EA to solidify and form a film. Figure 15 shows the formation of peptide bonds and disulfide bonds. Disulfide bond formation is an irreversible process and is responsible for the thermally cross-linked solid albumen film [43]. The denaturation of the proteins changes the oxygen diffusion paths and reduces the probability of oxygen scattering, which results in an increased possibility of conductive channels forming and rupturing in bio-memristors [16]. The resistance switching mechanism of the ITO/GO/EA/GO/Al devices is illustrated in Figure 16. When the glass/ITO/GO/EA/GO/Al devices are not subjected to an applied bias, the initial state of the devices is the HRS. When the bottom electrode (ITO) of the device is grounded and a negative voltage is applied to the top electrode (Al) of the device, the oxygen ions in the EA film and the oxygen ions in the two GO film layers migrate toward the bottom electrode (ITO) under the action of the electric field, which leaves oxygen vacancies. With increasing negative voltage, the oxygen vacancies gradually increase. When the voltage reaches Vset, a conductive channel is formed by oxygen vacancies between the top and bottom electrodes. At this time, the resistance of the device sharply decreases, and the device changes from the HRS to the LRS. When a positive voltage is applied to the top electrode (Al) of the device, the oxygen ions in the EA film and the oxygen ions in the two GO film layers migrate toward the top electrode (Al) under the action of the electric field and simultaneously fill the oxygen vacancies. By increasing the positive voltage, the oxygen vacancies gradually decrease. When the voltage reaches Vreset, the conductive channel formed by oxygen vacancies between the top and bottom electrodes is broken, and the device switches from the LRS to the HRS. Under this mechanism, the device can realize bistable switching. The experimental results show that the resistance of the glass/ITO/EA/Al device is large in the HRS. When the two GO layers are introduced, the resistance of the glass/ITO/GO/EA/GO/Al devices in the HRS decreases, and the ON/OFF current ratio decreases. This is due to the migration of oxygen ions in the oxygen-containing functional groups of GO, which promotes the formation of conductive channels in the device. With increasing GO concentration, the oxygen-containing functional groups in the device increase, which forms more oxygen vacancy conductive channels and reduces the resistance of the device in the HRS. Therefore, the current switching ratio of the device decreases with an increasing GO concentration.

## 4. Conclusions

We prepared ITO/GO/EA/GO/Al devices with GO concentrations of 0.05 ωt %, 0.5 ωt %, and 2 ωt % in the GO layers and an ITO/EA/Al device. As the GO concentration decreases, the ON/OFF current ratio gradually increases, and Vreset decreases. In other words, by changing the GO concentration, the current switching ratio and threshold voltage can be adjusted. When the GO concentration is 0.5 ωt %, the maximum number of switching cycles of the device is more than 200. The endurance and uniformity are improved compared to those of the other samples. Lastly, we investigated the conduction mechanism of these devices and found that their resistance switching can be attributed to the formation and rupture of conductive channels.

## References

[1] Raeis-Hosseini N., Lee J.-S. (2016). Controlling the Resistive Switching Behavior in Starch-Based Flexible Biomemristors. ACS Appl. Mater. Interfaces.

[2] Mirkin C.A., Letsinger R.L., Mucic R.C., Storhoff J.J. (1996). A DNA-based method for rationally assembling nanoparticles into macroscopic materials. Nature.

[3] Qi Y., Sun B., Fu G., Li T., Zhu S., Zheng L. (2019). A nonvolatile organic resistive switching memory based on lotus leaves. Chem. Phys..

[4] Sivkov A.A., Xing Y., Cheong K.Y. (2020). Investigation of honey thin film as a resistive switching material for nonvolatile memories. Mater. Lett..

[5] Güzel R., Ocak Y.S., Karuk S.N. (2019). Light harvesting and photo-induced electrochemical devices based on bionanocage proteins. J. Power Sour..

[6] Yukimoto T., Uemura S., Kamata T. (2011). Non-volatile transistor memory fabricated using DNA and eliminating influence of mobile ions on electric properties. J. Mater. Chem..

[7] Chang J.W., Wang C.G., Huang C.Y. (2011). Chicken Albumen Dielectrics in Organic Field-Effect Transistors. Adv. Mater..

[8] Zhu Z., Tam M.T., Sun F. (2014). A high-energy-density sugar biobattery based on a synthetic enzymatic pathway. Nat. Commun..

[9] Gomez E.F., Venkatraman V., Grote J.G. (2014). DNA Bases Thymine and Adenine in Bio-Organic Light Emitting Diodes. Sci. Rep..

[10] Zheng L., Sun B., Chen Y. (2018). The redox of hydroxyl-assisted metallic filament induced resistive switching memory based on a biomaterial-constructed sustainable and environment-friendly device. Mater. Today Chem..

[11] Zhou G., Sun B., Zhou A. (2017). A larger nonvolatile bipolar resistive switching memory behaviour fabricated using eggshells. Curr. Appl. Phys..

[12] Abbas Y., Dugasani S.R., Raza M.T. (2019). The observation of resistive switching characteristics using transparent and biocompatible Cu^2+^-doped salmon DNA composite thin film. Nanotechnology.

[13] Celano U., Nagashima K., Koga H., Nogi M., Zhuge F., Meng G., He Y., Boeck J.D., Jurczak M., Vandervorst W. (2016). All-nanocellulose nonvolatile resistive memory. Npg Asia Mater..

[14] Wang H., Meng F., Ca Y., Zheng L., Li Y., Liu Y., Jiang Y., Wang X., Chen X. (2013). Sericin for resistance switching device with multilevel nonvolatile memory. Adv. Mater..

[15] Mukherjee C., Hota M.K., Naskar D. (2013). Resistive switching in natural silk fibroin protein-based bio-memristors. Physica Status Solidi (a).

[16] Bok C.H., Woo S.J., Wu C. (2017). Flexible bio-memristive devices based on chicken egg albumen:Au@SiO_2_ core-shell nanoparticle nanocomposites. Sci. Rep..

[17] Park Y., Lee J.-S. (2017). Artificial Synapses with Short- and Long-Term Memory for Spiking Neural Networks Based on Renewable Materials. Acs Nano.

[18] Raeis-Hosseini N., Park Y., Lee J.-S. (2018). Flexible Artificial Synaptic Devices Based on Collagen from Fish Protein with Spike-Timing-Dependent Plasticity. Adv. Funct. Mater..

[19] Yang Y., Lu W. (2013). Nanoscale resistive switching devices: Mechanisms and modeling. Nanoscale.

[20] Celano U., Goux L., Degraeve R., Fantini A., Richard O., Bender H., Jurcza M., Vandervorst W. (2015). Imaging the three-dimensional conductive channel in filamentary-based oxide resistive switching memory. Nano Lett..

[21] Chandrakishore S., Pandurangan A. (2014). Facile synthesis of carbon nanotubes and their use in the fabrication of resistive switching memory devices. Rsc Adv..

[22] Avila-Nino J.A., Machado W.S., Sustaita A.O. (2012). Organic low voltage rewritable memory device based on PEDOT:PSS/f-MWCNTs thin film. Org. Electron..

[23] Chaoxing W., Li F., Zhang Y. (2011). Highly reproducible memory effect of organic multilevel resistive-switch device utilizing graphene oxide sheets/polyimide hybrid nanocomposite. Appl. Phys. Lett..

[24] Murgunde B.K., Rabinal M.K. (2017). Solution processed bilayer junction of silk fibroin and semiconductor quantum dots as multilevel memristor devices. Org. Electron..

[25] Thakre A., Borkar H., Singh B.P., Kumar A. (2015). Electroforming free high resistance resistive switching of graphene oxide modified polar-PVDF. Rsc Adv..

[26] Zhang B., Chen Y., Ren Y. (2013). In Situ Synthesis and Nonvolatile Rewritable-Memory Effect of Polyaniline-Functionalized Graphene Oxide. Chemistry.

[27] Yang P., Ma X., Ni X. (2016). Nonvolatile resistance switching memory devices fabricated from the photopolymerized poly(N-vinylcarbazole)-graphene oxide composites. J. Mater. Sci. Mater. Electron..

[28] Choi J.-Y., Yu H.-C., Lee J. (2018). Preparation of Polyimide/Graphene Oxide Nanocomposite and Its Application to Nonvolatile Resistive Memory Device. Polymers.

[29] Kim T., Kim D.-K., Kim J. (2019). Resistive switching behaviour of multi- stacked PVA/graphene oxide + PVA composite/PVA insulating layer-based RRAM devices. Semicond. Sci. Technol..

[30] Zhang B., Liu L., Wang L. (2018). Covalent Modification of Graphene Oxide with Poly(N-vinylcarbazole) Containing Pendant Azobenzene Chromophores for Nonvolatile Ternary memories. Carbon.

[31] Jesuraj P.J., Parameshwari R., Jeganathan K. (2018). Improved performance of graphene oxide based resistive memory devices through hydrogen plasma. Mater. Lett..

[32] Khurana G., Misra P., Katiyar R.S. (2013). Forming free resistive switching in graphene oxide thin film for thermally stable nonvolatile memory applications. J. Appl. Phys..

[33] Jeong H.Y., Kim J.Y., Kim J.W. (2010). Graphene Oxide Thin Films for Flexible Nonvolatile Memory Applications. Nano Lett..

[34] Kim I., Siddik M., Shin J. (2011). Low temperature solution-processed graphene oxide/Pr_0.7_Ca_0.3_MnO_3_ based resistive-memory device. Appl. Phys. Lett..

[35] Khurana G., Misra P., Kumar N. (2014). Tunable Power Switching in Nonvolatile Flexible Memory Devices Based on Graphene Oxide Embedded with ZnO Nanorods. J. Phys. Chem. C.

[36] Pradhan S.K., Xiao B., Mishra S., Killam A., Pradhan A.K. (2016). Resistive switching behavior of reduced graphene oxide memory cells for low power nonvolatile device application. Sci. Rep..

[37] Sharma S.K., Prakash J., Pujari P.K. (2015). Effects of the molecular level dispersion of graphene oxide on the free volume characteristics of poly (vinyl alcohol) and its impact on the thermal and mechanical properties of their nanocomposites. Phys. Chem. Chem. Phys..

[38] Zhu C., Mahmood Z., Zhang W., Akram M.W., Ainur D., Ma H. (2020). In situ investigation of acute exposure of graphene oxide on activated sludge: Biofilm characteristics, microbial activity and cytotoxicity. Ecotoxicol. Environ. Saf..

[39] Banerjee I., Harris P., Salimian A. (2015). Graphene oxide thin films for resistive memory switches. Let Circuits Devices Syst..

[40] Li L., Li G. (2020). Multi-Bit biomemory based on chitosan: Graphene oxide nanocomposite with wrinkled surface. Micromachines.

[41] Alves A.K., Frantz A.C.S., Berutti F.A. (2018). Microwave-assisted oleothermal synthesis of graphene-TiO_2_ quantum dots for photoelectrochemical oxygen evolution reaction. Flatchem.

[42] Soliva-Fortuny R., Balasa A., Knorr D. (2009). Effects of pulsed electric fields on bioactive compounds in foods: A review. Trends Food Sci. Technol..

[43] He X.L., Zhang J., Wang W.B., Xuan W.P., Wang X.Z., Zhang Q.L., Smith C.G., Luo J.K. (2016). Transient resistive switching devices made from egg albumen dielectrics and dissolvable electrodes. Acs Appl. Mater. Interfaces.

